# 

**Published:** 1790

**Authors:** 


### CONTENTS.

Page

I. *A Case of Retention of Urine, in which a Puncture of the Bladder, in the hypogastric Region, proved, under very unpromising Circumstances, successful; to which are added three Cases of Retention of Urine, and some Remarks on Diseases of the urinary Bladder. By Mr.* James Lucas, *one of the Surgeons to the General Infirmary at Leeds.* 109

II. *An Account of the singular Effects of Music on a Patient. By the same.* 125

III. *A Case of fractured Ribs which terminated fatally; with the Appearances on Dissection, and Remarks. By Mr.* George Wilkinson, *Surgeon at Sunderland, Member of the Royal College of Surgeons, and Honorary Member of the Chirurgo-physical Society of Edinburgh.* — — — 130

IV. *Case of an Abscess in the lower Part of the Belly which communicated with the Intestine, and terminated successfully. By Mr.* George Grant, *Surgeon.* — — 138

V. *Extract of a Letter from Mr.* Philip Werner, *Surgeon of the Royal Navy, and of the British Factory at Algiers, to Dr.* Simmons, *containing some Account of the Inoculation of the Small Pox in Algiers; together with other miscellaneous Observations.* — 140

VI. *Case of a Woman who, after having been gored in the Abdomen by an Ox in the sixth Month of Pregnancy, underwent the Cæ-*

Page

*ſarean Operation.* *By* Frederick Auguſtus Fritſe, *M. D. Phyſician at Dillenburgh.* 146

VII. *An Account of the Effects of Laurel Water as obſerved in the Bodies of two Perſons who died at Turin, January* 22, 1785. *By M.* Penchienati, *Member of the Royal Academy of Sciences at Turin.* — 160

VIII. *Obſervations on Gangrenes and Mortifications, accompanied with, or occaſioned by, convulſive Spaſms, or ariſing from local Injury, producing Irritation.* *By* Charles White, *Eſq. F. R. S. Surgeon to the Infirmary and Lunatic Hoſpital, and Vice-Preſident of the Literary and Philoſophical Society at Mancheſter,* &c. —— —— 167

IX. *Pathological Obſervations on the Brain.* *By Mr.* Thomas Anderſon, *F. R. S. Edin. Surgeon at Leith, and Fellow of the Royal College of Surgeons.* — *From The Tranſactions of the Royal Society of Edinburgh.* —— 182

X. *An Account of a Diſtemper, by the common People in England vulgarly called the Mumps.* *By* Robert Hamilton, *M. D. Fellow of the Royal College of Phyſicians, F. R. S. Edin. and Phyſician at Lynn Regis, Norfolk.* — *From The Tranſactions of the Royal Society of Edinburgh.* —— — 190

Catalogue *of* Books. — 211

